# Supplementary material for: Resurrection of plant disease resistance proteins via helper NLR bioengineering
Source: Sci Adv. 2023 May 3;9(18):eadg3861. doi: 10.1126/sciadv.adg3861 (PMC10156107; doi:10.1126/sciadv.adg3861)
Supplement: Supplementary file 1 — Figs. S1 to S9 Legends for tables S1 and S2 Table S3 Legend for movie S1 References [file sciadv.adg3861_sm.pdf]

Science Advances
AAAS

Supplementary Materials for

Resurrection of plant disease resistance proteins via helper NLR bioengineering

Mauricio P. Contreras *et al.*

Corresponding author: Sophien Kamoun, sophien.kamoun@tsl.ac.uk; Lida Derevnina, ld645@cam.ac.uk

Sci. Adv. **9**, eadg3861 (2023)
DOI: 10.1126/sciadv.adg3861

The PDF file includes:

Figs. S1 to S9
Legends for tables S1 and S2
Table S3
Legend for movie S1
References

Other Supplementary Material for this manuscript includes the following:

Tables S1 and S2
Movie S1

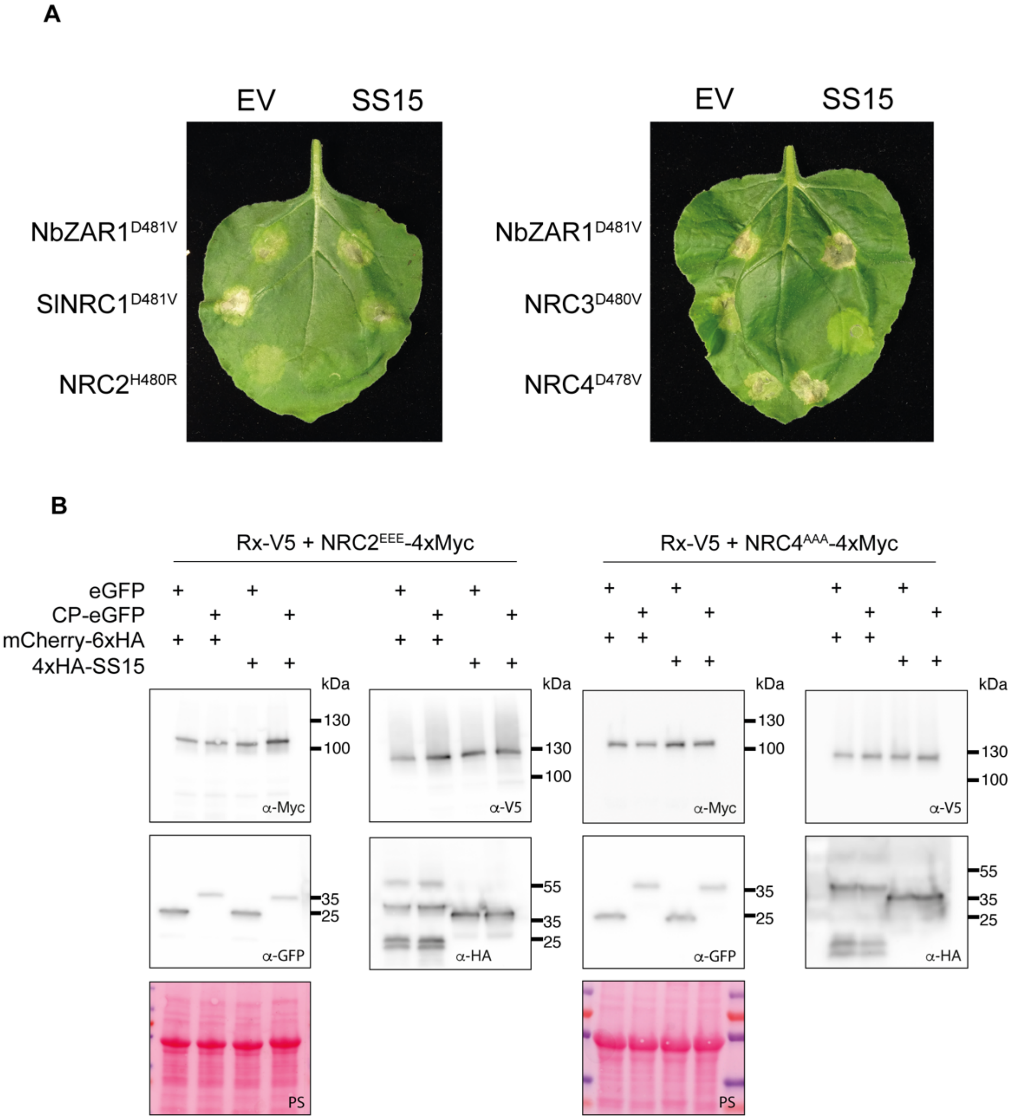
A
EV
SS15
NbZAR1D481V
SlNRC1D481V
NRC2H480R
EV
SS15
NbZAR1D481V
NRC3D480V
NRC4D478V
B
Rx-V5 + NRC2EEE-4xMyc
Rx-V5 + NRC4AAA-4xMyc
eGFP
CP-eGFP
mCherry-6xHA
4xHA-SS15
kDa
130
100
α-Myc
α-V5
35
25
55
α-GFP
α-HA
PS

Fig. S1: SS15 suppresses cell death mediated by SlNRC1, NRC2 and NRC3 but not NRC4 or NbZAR1. (A) Photo of representative leaves from *N. benthamiana nrc2/3/4* KO plants showing HR after co-expression of various autoactive NLR variants with a free mCherry-6xHA fusion protein (EV) or with N-terminally 4xHA-tagged SS15. Images are representative of **(B)** SDS-PAGE accompanying BN-PAGE shown in Fig. 1B. Total protein extracts were immunoblotted with the appropriate antisera labelled on the left. Approximate molecular weights (kDa) of the proteins are shown on the right. Rubisco loading control was carried out using Ponceau stain (PS). The experiment was repeated three times with similar results.

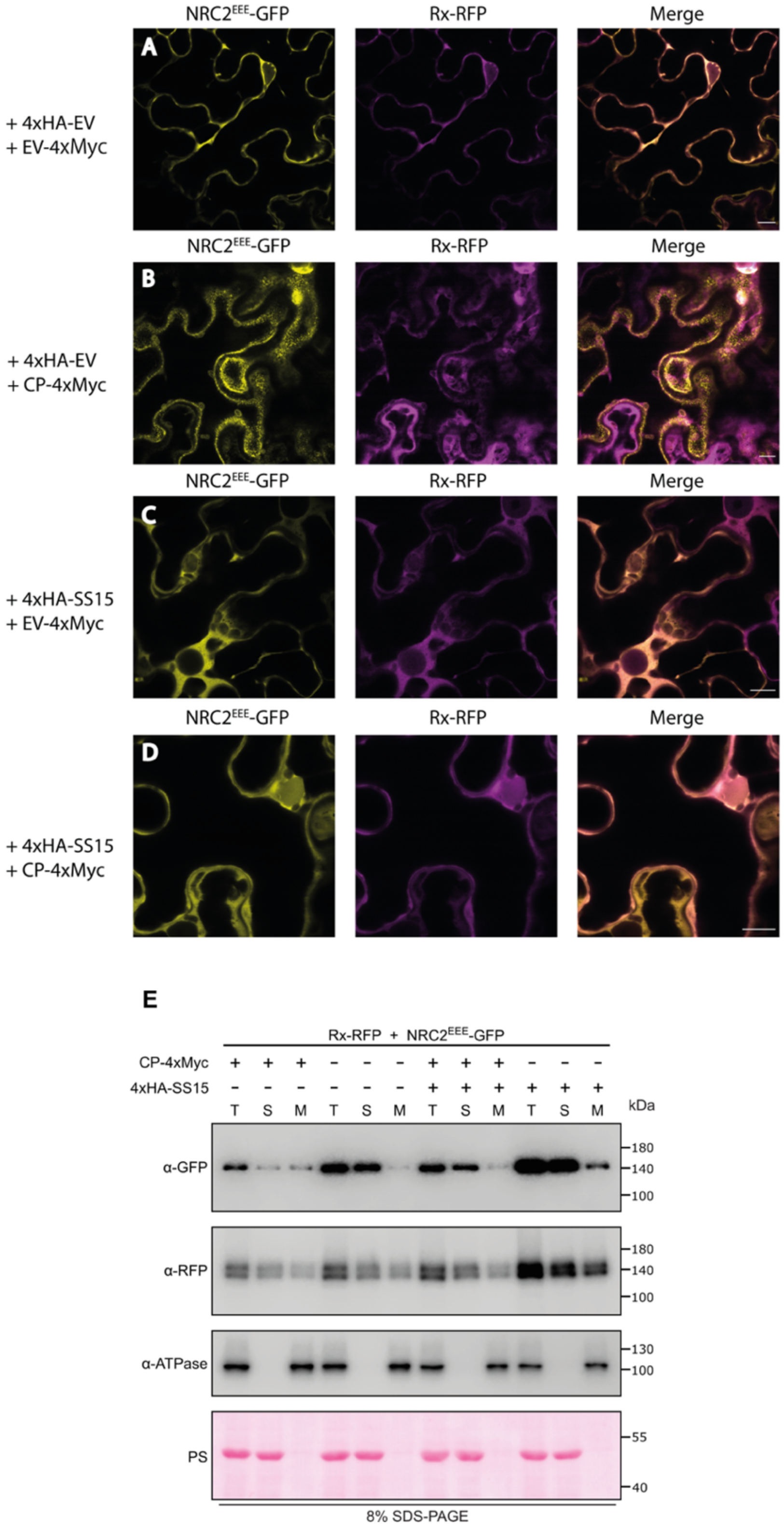
NRC2^{EEE}-GFP
Rx-RFP
Merge
A
B
C
D
+ 4xHA-EV
+ EV-4xMyc
+ 4xHA-EV
+ CP-4xMyc
+ 4xHA-SS15
+ EV-4xMyc
+ 4xHA-SS15
+ CP-4xMyc
E
Rx-RFP + NRC2^{EEE}-GFP
CP-4xMyc
4xHA-SS15
T S M T S M T S M T S M
kDa
α-GFP
α-RFP
α-ATPase
PS
180
140
100
130
55
40
8% SDS-PAGE

Fig. S2: SS15 inhibits plasma membrane-association of activated NRC2. (**A-D**) C-terminally GFP-tagged NRC2^{EEE} and C-terminally RFP-tagged Rx were co-expressed with an EV-4xMyc construct or a CP-4xMyc construct in leaves of *nrc2/3/4* KO *N. benthamiana*. Representative single-plane confocal micrographs show the localization of both components of the inactive and active Rx-NRC2 system. Scale bars represent 10 μm. (**A**) NRC2^{EEE}-GFP and Rx-RFP co-localize in the cytoplasm. (**B**) As reported previously, Rx/CP activated NRC2^{EEE} forms plasma membrane-associated puncta while Rx remains in the cytoplasm. (**C**) Co-expression with SS15 does not alter the localization of inactive NRC2^{EEE}-GFP or Rx-RFP. (**D**) Upon co-expression CP-4xMyc, Rx-RFP and NRC2^{EEE}-GFP with SS15, the punctate localization for NRC2^{EEE}-GFP is no longer observed. (**E**) Membrane enrichment assays are consistent with microscopy. As reported previously, inactive NRC2^{EEE}-GFP is mostly present in the soluble fraction (S) whereas activated NRC2^{EEE}-GFP exhibits equal distribution across soluble and membrane (M) fractions. Upon co-expression with SS15, NRC2^{EEE}-GFP distribution remains in the soluble fraction regardless of the presence or absence of PVX CP. The experiment was repeated twice with similar results.

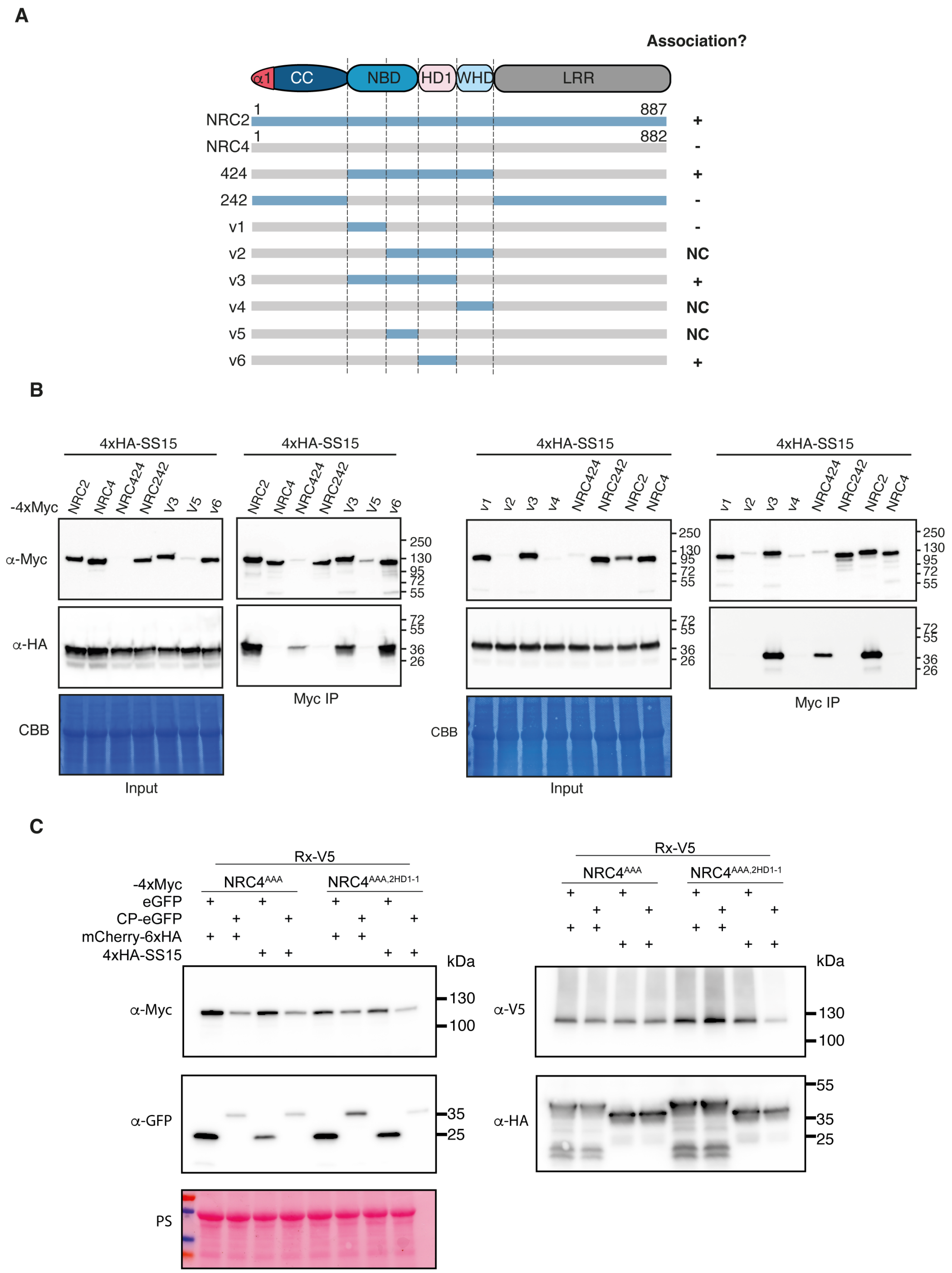

A
Association?
α1
CC
NBD
HD1
WHD
LRR
NRC2
1
887
+
NRC4
1
882
-
424
+
242
-
v1
-
v2
NC
v3
+
v4
NC
v5
NC
v6
+
B
4xHA-SS15
-4xMyc
NRC2
NRC4
NRC424
NRC242
V3
V5
v6
v1
v2
v3
v4
α-Myc
α-HA
CBB
Input
Myc IP
250
130
95
72
55
36
26
C
Rx-V5
-4xMyc
NRC4AAA
NRC4AAA,2HD1-1
eGFP
CP-eGFP
mCherry-6xHA
4xHA-SS15
kDa
α-Myc
130
100
α-GFP
35
25
PS
α-V5
α-HA
55

Fig. S3: SS15 inhibits NRC2 by interacting with the HD1-1 region of the NB-ARC domain.

(A) Schematic representation of all NRC2-NRC4 chimeric proteins generated. Association with SS15 (+) or lack thereof (-) is indicated on the right. (**B**) Co-Immunoprecipitation (Co-IP) assays between SS15 and chimeric NRC2-NRC4 variants. C-terminally 4xMyc-tagged NRC proteins were transiently co-expressed with N-terminally 4xHA-tagged SS15. IPs were performed with agarose beads conjugated to Myc antibodies (Myc IP). Total protein extracts were immunoblotted with the appropriate antisera labelled on the left. Approximate molecular weights (kDa) of the proteins are shown on the right. Rubisco loading control was carried out using Ponceau stain (PS). The experiment was repeated three times with similar results. (**C**) SDS-PAGE accompanying BN-PAGE shown in **Fig. 2E**. Total protein extracts were immunoblotted with the appropriate antisera labelled on the left. Approximate molecular weights (kDa) of the proteins are shown on the right. Rubisco loading control was carried out using Ponceau stain (PS). The experiment was repeated three times with similar results.

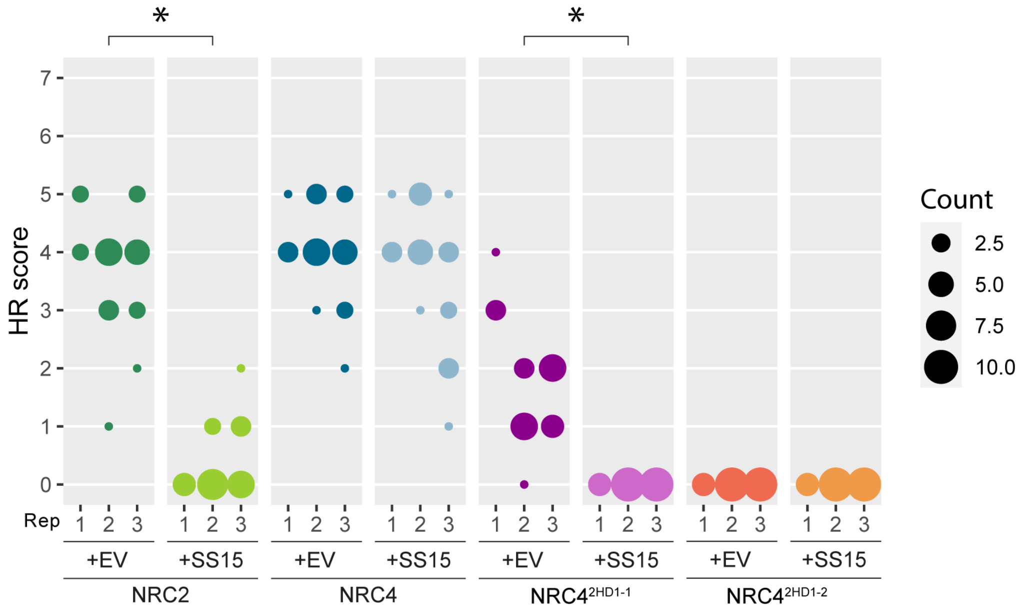

Fig. S4: NRC4^{HD1-1} chimera is susceptible to inhibition by SS15. HR scores accompanying Fig. 2D. In all cases, Rx/CP was used to activate the system. HR was scored based on a modified 0–7 scale (*49*) between 5–7 days post-infiltration. HR scores are presented as dot plots, where the size of each dot is proportional to the number of samples with the same score (Count). Results are based on 3 biological replicates. Statistical tests were implemented using the besthr R library (*50*). We performed bootstrap resampling tests using a lower significance cut-off of 0.025 and an upper cut-off of 0.975. Mean ranks of test samples falling outside of these cut-offs in the control samples bootstrap population were considered significant. Significant differences between the conditions are indicated with an asterisk (*).

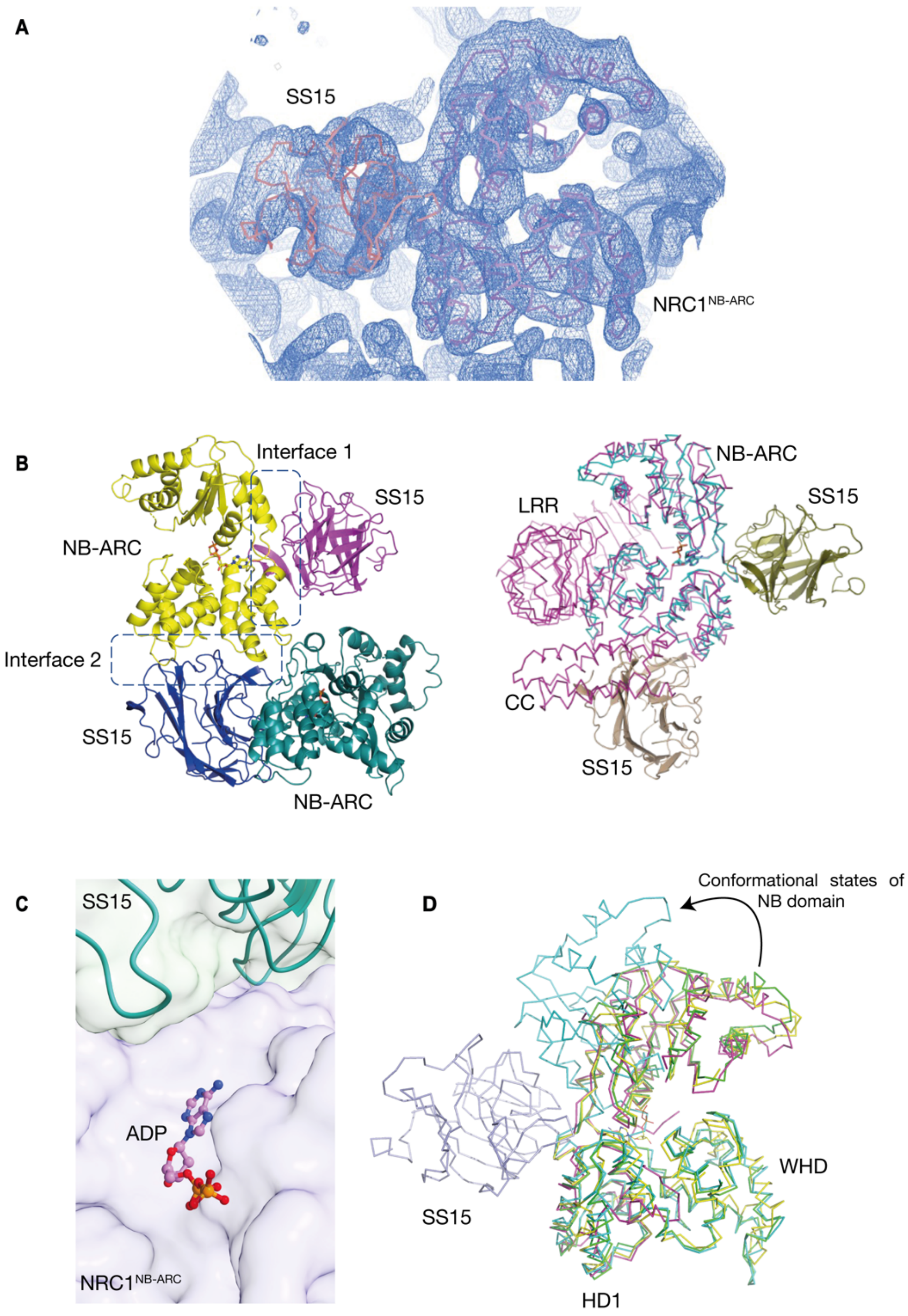
A
SS15
NRC1NB-ARC
B
Interface 1
SS15
NB-ARC
Interface 2
SS15
NB-ARC
NB-ARC
LRR
SS15
CC
SS15
C
SS15
ADP
NRC1NB-ARC
D
Conformational states of NB domain
WHD
SS15
HD1

Fig. S5: Crystal structure of SS15 in complex with SlNRC1$^{NB\text{-}ARC}$. Electron density map showing the relative orientation and arrangement of SS15 (orange) and SlNRC1$^{NB\text{-}ARC}$ (violet) within an asymmetric unit. 2Fo-Fc map countered at 1σ **(B)** Two possible interfaces between SS15 and SlNRC1$^{NB\text{-}ARC}$ revealed from the crystal packing. Both interfaces (Interface 1 and Interface 2) are outlined (Left). Modelling of both potential binding interfaces for SS15 complex with full length SlNRC1 (magenta) reveals a steric clash between the CC-domain of SlNRC1 and SS15, making interface 2 unlikely to be biologically relevant in the full-length context (Right). **(C)** Close up view of interaction between SS15-SlNRC1$^{NB\text{-}ARC}$ interaction interface relative to the ATP-binding site within the NB-ARC domain of SlNRC1. The pyrophosphate moiety of ADP is oriented facing opposite the SS15 binding interface (shown as ball and sticks), suggesting that SS15 is unlikely to displace bound nucleotide or prevent ATP hydrolysis. **(D)** Structure of SS15-SlNRC1$^{NB\text{-}ARC}$ (yellow, PDB 8BV0) is superimposed over the NB-ARC domain of AtZAR1 in its inactive (green, PDB 6J5W), intermediate (cyan, PDB 6J5V), and active resistosome (magenta, 6J5T) conformations. Visualizing these three states reveals the trajectory of the NB domain as it moves relative to the HD1 and WHD domains while changing from inactive to activated states. The binding of SS15 at the critical hinge region between the NB and HD1-WHD domains likely immobilizes this loop, preventing these critical intramolecular rearrangements and therefore preventing NLR activation. See **Movie S1**.

A

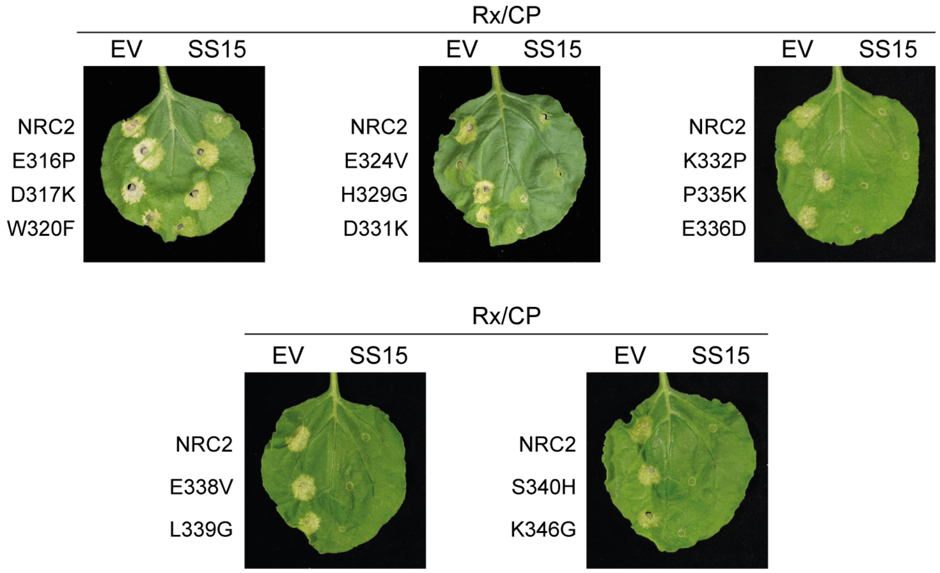
Rx/CP
EV
SS15
NRC2
E316P
D317K
W320F
E324V
H329G
D331K
K332P
P335K
E336D
E338V
L339G
S340H
K346G

B

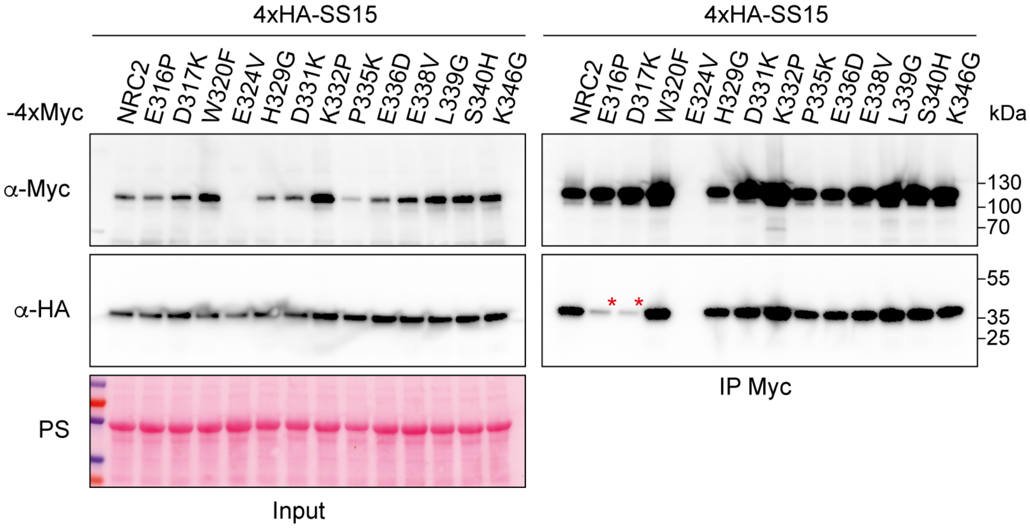
4xHA-SS15
-4xMyc
NRC2
E316P
D317K
W320F
E324V
H329G
D331K
K332P
P335K
E336D
E338V
L339G
S340H
K346G
kDa
α-Myc
α-HA
PS
130
100
70
55
35
25
Input
IP Myc

Fig. S6: Out of 13 NRC2 variants tested, only E316P and D317K mutations abolish SS15 association and HR suppression. (A) Photo of representative leaves from *N. benthamiana nrc2/3/4* KO plants showing HR after co-expression of Rx and PVX CP with NRC2, or the different NRC2 variants generated. These effector-sensor-helper combinations were co-expressed with a free mCherry-6xHA fusion protein (EV) or with N-terminally 4xHA-tagged SS15. (**B**) Co-Immunoprecipitation (Co-IP) assays between SS15 and NRC2 variants. C-terminally 4xMyc-tagged NRC2 variants were transiently co-expressed with N-terminally 4xHA-tagged SS15. IPs were performed with agarose beads conjugated to Myc antibodies (Myc IP). Total protein extracts were immunoblotted with appropriate antisera labelled on the left. Approximate molecular weights (kDa) of the proteins are shown on the right. Rubisco loading control was carried out using Ponceau stain (PS). The experiment was repeated three times with similar results.

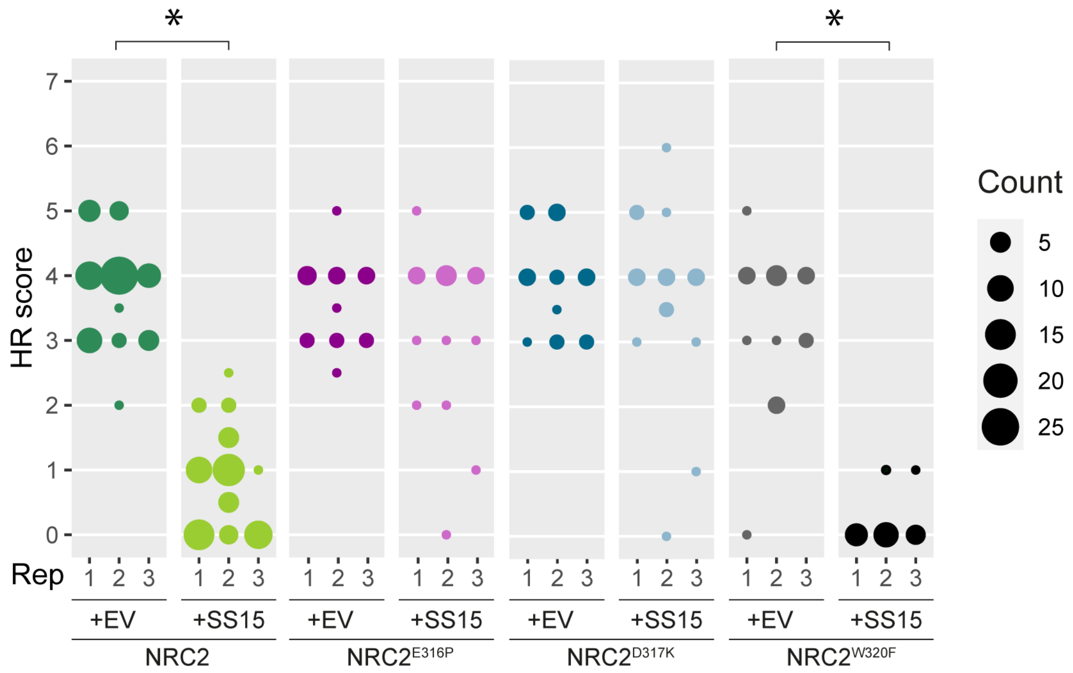

Fig. S7: NRC2^{E316P} and NRC2^{D317K} abolish SS15-mediated suppression of Rx.
HR scores accompanying **Fig. 3C**. In all cases, Rx/CP was used to activate the system. HR was scored based on a modified 0–7 scale (*49*) between 5–7 days post-infiltration. HR scores are presented as dot plots, where the size of each dot is proportional to the number of samples with the same score (Count). Results are based on 3 biological replicates. Statistical tests were implemented using the besthr R library (*50*). We performed bootstrap resampling tests using a lower significance cut-off of 0.025 and an upper cut-off of 0.975. Mean ranks of test samples falling outside of these cut-offs in the control samples bootstrap population were considered significant. Significant differences between the conditions are indicated with an asterisk (*).

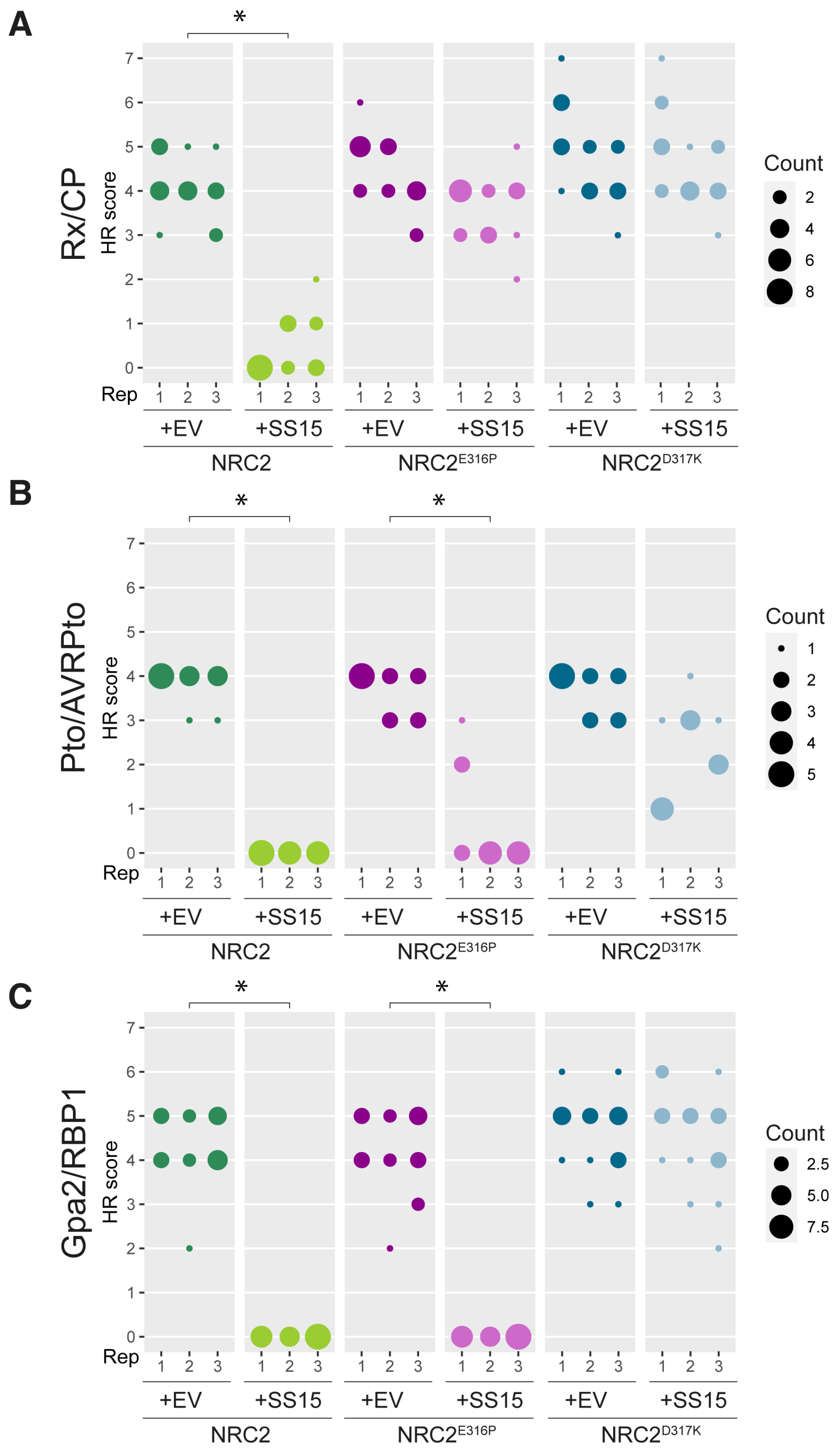

A
Rx/CP
HR score
Count
2
4
6
8
B
Pto/AVRPto
Count
1
2
3
4
5
C
Gpa2/RBP1
Count
2.5
5.0
7.5
Rep
+EV
+SS15
NRC2
NRC2E316P
NRC2D317K

Fig. S8: NRC2^{D317K} abolishes SS15-mediated suppression of Rx, Gpa2 and Prf.
HR scores accompanying **Fig. 4A**. NRCs were activated using Rx/CP (**A**), Pto/AVRPto (**B**) or Gpa2/RBP1 (**C**). HR was scored based on a modified 0–7 scale (*49*) between 5–7 days post-infiltration. HR scores are presented as dot plots, where the size of each dot is proportional to the number of samples with the same score (count). Results are based on 3 biological replicates. Statistical tests were implemented using the besthr R library (*50*). We performed bootstrap resampling tests using a lower significance cut-off of 0.025 and an upper cut-off of 0.975. Mean ranks of test samples falling outside of these cut-offs in the control samples bootstrap population were considered significant. Significant differences between the conditions are indicated with an asterisk (*).

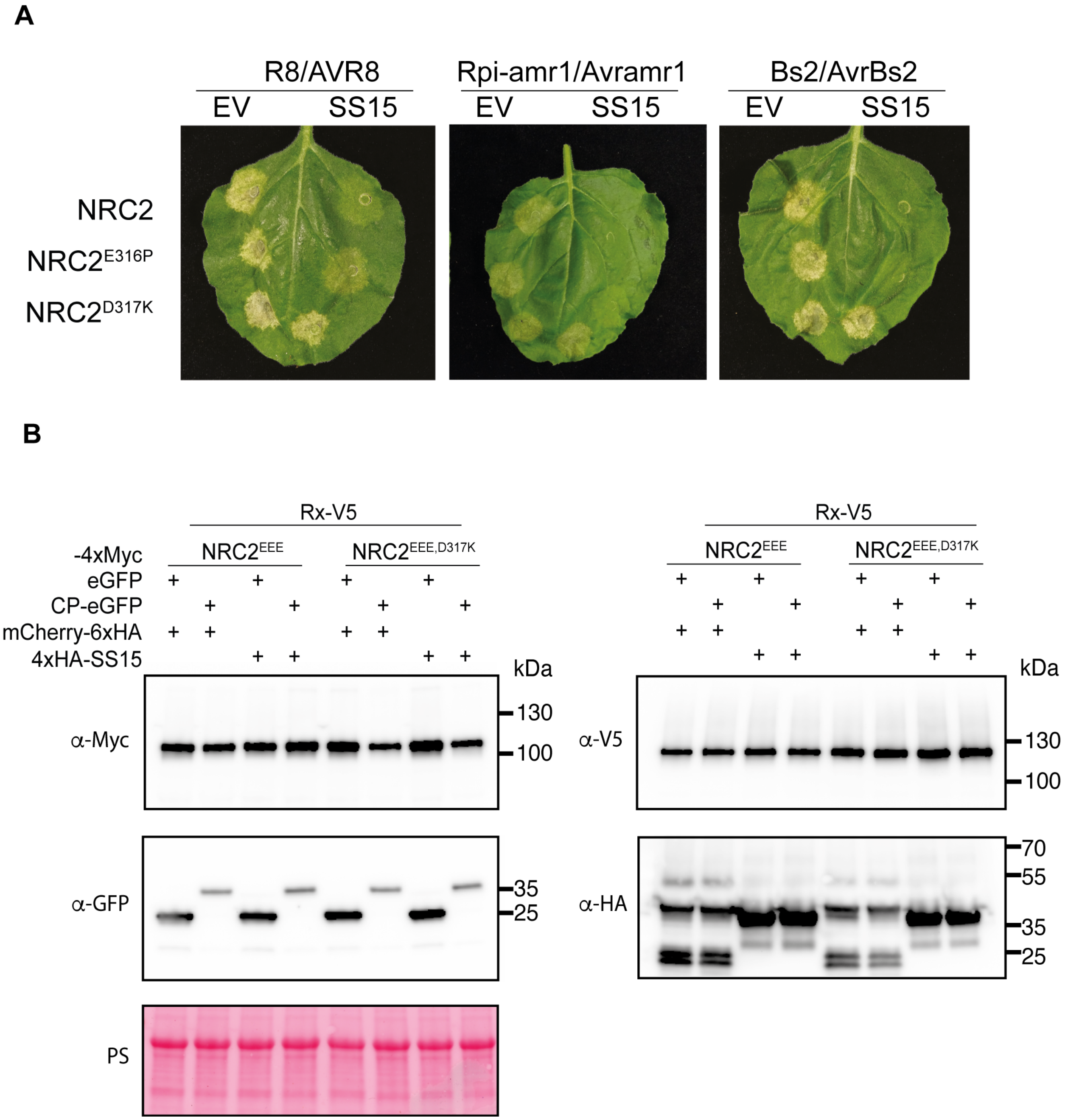
A
R8/AVR8
EV
SS15
Rpi-amr1/Avramr1
EV
SS15
Bs2/AvrBs2
EV
SS15
NRC2
NRC2E316P
NRC2D317K
B
Rx-V5
-4xMyc
NRC2EEE
NRC2EEE,D317K
eGFP
CP-eGFP
mCherry-6xHA
4xHA-SS15
kDa
α-Myc
130
100
α-GFP
35
25
PS
Rx-V5
NRC2EEE
NRC2EEE,D317K
kDa
α-V5
130
100
α-HA
70
55
35
25

Fig. S9: NRC2^{D317K} abolishes SS15-mediated suppression of all NRC2-dependent sensors tested and restores NRC2 resistosome formation. Photo of representative leaves from *N. benthamiana nrc2/3/4* KO plants showing HR after co-expression of NRC2, or different NRC2 variants generated with various sensor/effector pairs. These effector-sensor-helper combinations were co-expressed with a free mCherry-6xHA fusion protein (EV) or with N-terminally 4xHA-tagged SS15. (**B**) SDS-PAGE accompanying BN-PAGE shown in **Fig. 4B**. Total protein extracts were immunoblotted with the appropriate antisera labelled on the left. Approximate molecular weights (kDa) of the proteins are shown on the right. Rubisco loading control was carried out using Ponceau stain (PS). The experiment was repeated three times with similar results.

Table S1: List of constructs used in this study. (As separate file)

Table S2: List of OD_{600} used for agroinfiltration experiments. (As separate file)

Table S3: Summary of X-ray data and model parameters for NRC1-SS15.

Data collection	
Diamond Light Source beamline	I03
Wavelength (Å)	0.9763
Detector	Eiger2 XE 16M
Resolution range (Å)	51.34 – 4.50 (5.03 – 4.50)
Space Group	$P6_1$
Cell parameters (Å)	$a = b = 128.6$, $c = 170.7$
Total no. of measured intensities	77102 (13865)
Unique reflections	8981 (2263)
Multiplicity	8.6 (6.1)
Mean $I/\sigma(I)$	7.2 (1.5)
Completeness (%)	94.0 (84.0)
R_{merge}[a]	0.084 (1.513)
R_{meas}[b]	0.033 (0.612)
$CC_{½}$[c]	0.998 (0.588)
Refinement	
Resolution range (Å)	51.34 – 4.50 (4.62 – 4.50)
Reflections: working/free[d]	8075/883
R_{work}/ R_{free}[e]	0.237/0.275
MolProbity score/Clashscore[f]	1.58/5.41
Ramachandran plot: favoured/allowed/disallowed[f] (%)	95.9/4.1/0.0
R.m.s. bond distance deviation (Å)	0.003
R.m.s. bond angle deviation (°)	0.79
NRC1 – chains/no. protein residues/ranges	A,C/343/153-494
SS15 – chains/no. protein residues/ranges	B,D/206/18-223
No. ADP molecules/RSCC[g]	2/0.72,0.84
PDB accession code	8BV0

Values in parentheses are for the outer resolution shell.

[a] $R_{merge} = \sum_{hkl} \sum_{I} |I_i(hkl) - \langle I(hkl)\rangle| / \sum_{hkl} \sum_i I_i(hkl)$.

[b] $R_{meas} = \sum_{hkl} [N/(N-1)]^{1/2} \times \sum_I |I_i(hkl) - \langle I(hkl) \rangle| / \sum_{hkl} \sum_i I_i(hkl)$, where $I_i(hkl)$ is the ith observation of reflection *hkl*, $\langle I(hkl) \rangle$ is the weighted average intensity for all observations I of reflection *hkl* and N is the number of observations of reflection *hkl*.

[c] $CC_{½}$ is the correlation coefficient between symmetry equivalent intensities from random halves of the dataset.

[d] The data set was split into “working” and “free” sets consisting of 90 and 10% of the data respectively. The free set was not used for refinement.

[e] The R-factors R_{work} and R_{free} are calculated as follows: $R = \sum(|F_{obs} - F_{calc}|)/\sum|F_{obs}|$, where F_{obs} and F_{calc} are the observed and calculated structure factor amplitudes, respectively.

[f] As calculated using MolProbity (*51*).

[g] Real Space Correlation Coefficient as calculated by the PDB validation server.

Movie S1: SS15 immobilizes hinge region between NB and HD1-WHD domains of NRCs to prevent NLR activation.

REFERENCES

1. J. D. Jones, R. E. Vance, J. L. Dangl, Intracellular innate immune surveillance devices in plants and animals. *Science* **354**, aaf6395 (2016).

2. Z. Duxbury, C.-H. Wu, P. Ding, A comparative overview of the intracellular guardians of plants and animals: NLRs in innate immunity and beyond. *Annu. Rev. Plant Biol.* **72**, 155–184 (2021).

3. J. Wang, M. Hu, J. Wang, J. Qi, Z. Han, G. Wang, Y. Qi, H. W. Wang, J. M. Zhou, J. Chai, Reconstitution and structure of a plant NLR resistosome conferring immunity. *Science* **364**, eaav5870 (2019).

4. M. P. Contreras, H. Pai, Y. Tumtas, C. Duggan, E. L. H. Yuen, A. V. Cruces, J. Kourelis, H.-K. Ahn, C.-H. Wu, T. O. Bozkurt, L. Derevnina, S. Kamoun, Sensor NLR immune proteins activate oligomerization of their NRC helper. bioRxiv 2022.04.25.489342 [**Preprint**]. 25 April 2022. https://doi.org/10.1101/2022.04.25.489342.

5. H.-K. Ahn, X. Lin, A. C. Olave-Achury, L. Derevnina, M. P. Contreras, J. Kourelis, S. Kamoun, J. D. G. Jones, Effector-dependent activation and oligomerization of NRC helper NLRs by Rpi-amr3 and Rpi-amr1. bioRxiv 2022.04.25.489359 [**Preprint**]. 25 April 2022. https://doi.org/10.1101/2022.04.25.489359.

6. A. Förderer, E. Li, A. Lawson, Ya-nan Deng, Y. Sun, E. Logemann, X. Zhang, J. Wen, Z. Han, J. Chang, Y. Chen, P. Schulze-Lefert, J. Chai, A wheat resistosome defines common principles of immune receptor channels. bioRxiv 2022.03.23.485489 [**Preprint**]. 23 March 2022. https://doi.org/10.1101/2022.03.23.485489.

7. R. Martin, T. Qi, H. Zhang, F. Liu, M. King, C. Toth, E. Nogales, B. J. Staskawicz, Structure of the activated ROQ1 resistosome directly recognizing the pathogen effector XopQ. *Science* **370**, eabd9993 (2020).

8. I. V. Hochheiser, M. Pilsl, G. Hagelueken, J. Moecking, M. Marleaux, R. Brinkschulte, E. Latz, C. Engel, M. Geyer, Structure of the NLRP3 decamer bound to the cytokine release inhibitor CRID3. *Nature* **604**, 184–189 (2022).

9. Z. Hu, Q. Zhou, C. Zhang, S. Fan, W. Cheng, Y. Zhao, F. Shao, H. W. Wang, S. F. Sui, J. Chai, Structural and biochemical basis for induced self-propagation of NLRC4. *Science* **350**, 399–404 (2015).

10. Z. Duxbury, S. Wang, C. I. MacKenzie, J. L. Tenthorey, X. Zhang, S. U. Huh, L. Hu, L. Hill, P. M. Ngou, P. Ding, J. Chen, Y. Ma, H. Guo, B. Castel, P. N. Moschou, M. Bernoux, P. N. Dodds, R. E. Vance, J. D. G. Jones, Induced proximity of a TIR signaling domain on a plant-mammalian NLR chimera activates defense in plants. *Proc. Natl. Acad. Sci. U.S.A.* **117**, 18832–18839 (2020).

11. B. P. M. Ngou, P. Ding, J. D. Jones, Thirty years of resistance: Zig-zag through the plant immune system. *Plant Cell* **34**, 1447–1478 (2022).

12. L. A. Gao, M. E. Wilkinson, J. Strecker, K. S. Makarova, R. K. Macrae, E. V. Koonin, F. Zhang, Prokaryotic innate immunity through pattern recognition of conserved viral proteins. *Science* **377**, eabm4096 (2022).

13. C.-H. Wu, L. Derevnina, S. Kamoun, Receptor networks underpin plant immunity. *Science* **360**, 1300–1301 (2018).

14. L. D. Cunha, J. M. Ribeiro, T. D. Fernandes, L. M. Massis, C. A. Khoo, J. H. Moffatt, H. J. Newton, C. R. Roy, D. S. Zamboni, Inhibition of inflammasome activation by *Coxiella burnetii* type IV secretion system effector IcaA. *Nat. Commun.* **6**, 10205 (2015).

15. L. Derevnina, M. P. Contreras, H. Adachi, J. Upson, A. Vergara Cruces, R. Xie, J. Skłenar, F. L. H. Menke, S. T. Mugford, D. MacLean, W. Ma, S. A. Hogenhout, A. Goverse, A. Maqbool, C. H. Wu, S. Kamoun, Plant pathogens convergently evolved to counteract redundant nodes of an NLR immune receptor network. *PLOS Biol.* **19**, e3001136 (2021).

16. H. Yen, N. Sugimoto, T. Tobe, Enteropathogenic Escherichia coli uses NleA to inhibit NLRP3 inflammasome activation. *PLOS Pathog.* **11**, e1005121 (2015).

17. Q. Chai, S. Yu, Y. Zhong, Z. Lu, C. Qiu, Y. Yu, X. Zhang, Y. Zhang, Z. Lei, L. Qiang, B. X. Li, Y. Pang, X. B. Qiu, J. Wang, C. H. Liu, A bacterial phospholipid phosphatase inhibits host pyroptosis by hijacking ubiquitin. *Science* **378**, eabq0132 (2022).

18. C. Marchal, H. Pai, S. Kamoun, J. Kourelis, Emerging principles in the design of bioengineered made-to-order plant immune receptors. *Curr. Opin. Plant Biol.* **70**, 102311 (2022).

19. J. Kourelis, T. Sakai, H. Adachi, S. Kamoun, RefPlantNLR is a comprehensive collection of experimentally validated plant disease resistance proteins from the NLR family. *PLOS Biol.* **19**, e3001124 (2021).

20. H. Adachi, L. Derevnina, S. Kamoun, NLR singletons, pairs, and networks: Evolution, assembly, and regulation of the intracellular immunoreceptor circuitry of plants. *Curr. Opin. Plant Biol.* **50**, 121–131 (2019).

21. C.-H. Wu, A. Abd-el-Haliem, T. O. Bozkurt, K. Belhaj, R. Terauchi, J. H. Vossen, S. Kamoun, NLR network mediates immunity to diverse plant pathogens. *Proc. Natl. Acad. Sci. U.S.A.* **114**, 8113–8118 (2017).

22. C. N. LaRock, B. T. Cookson, The Yersinia virulence effector YopM binds caspase-1 to arrest inflammasome assembly and processing. *Cell Host Microbe* **12**, 799–805 (2012).

23. H. Chen, D. Yang, F. Han, J. Tan, L. Zhang, J. Xiao, Y. Zhang, Q. Liu, The bacterial T6SS effector EvpP prevents NLRP3 inflammasome activation by inhibiting the $Ca2^{+}$-dependent MAPK-Jnk pathway. *Cell Host Microbe* **21**, 47–58 (2017).

24. H. S. Karki, S. Abdullah, Y. Chen, D. A. Halterman, Natural genetic diversity in the potato resistance gene RB confers suppression avoidance from Phytophthora effector IPI-O4. *Mol. Plant Microbe Interact.* **34**, 1048–1056 (2021).

25. H. Adachi, M. P. Contreras, A. Harant, C. H. Wu, L. Derevnina, T. Sakai, C. Duggan, E. Moratto, T. O. Bozkurt, A. Maqbool, J. Win, S. Kamoun, An N-terminal motif in NLR immune receptors is functionally conserved across distantly related plant species. *eLife* **8**, e49956 (2019).

26. J. Kourelis, M. P. Contreras, A. Harant, H. Adachi, L. Derevnina, C.-H. Wu, S. Kamoun, The helper NLR immune protein NRC3 mediates the hypersensitive cell death caused by the cell-surface receptor Cf-4. bioRxiv 2021.09.28.461843 [**Preprint**]. 28 September 2021. https://doi.org/10.1101/2021.09.28.461843.

27. J. Wang, J. Wang, M. Hu, S. Wu, J. Qi, G. Wang, Z. Han, Y. Qi, N. Gao, H. W. Wang, J. M. Zhou, J. Chai, Ligand-triggered allosteric ADP release primes a plant NLR complex. *Science* **364**, eaav5868 (2019).

28. J. Kourelis, C. Marchal, S. Kamoun, NLR immune receptor-nanobody fusions confer plant disease resistance. bioRxiv 2021.10.24.465418 [**Preprint**]. 24 October 2021. https://doi.org/10.1101/2021.10.24.465418.

29. E. Weber, C. Engler, R. Gruetzner, S. Werner, S. Marillonnet, A modular cloning system for standardized assembly of multigene constructs. *PLOS ONE* **6**, e16765 (2011).

30. C. Engler, M. Youles, R. Gruetzner, T. M. Ehnert, S. Werner, J. D. G. Jones, N. J. Patron, S. Marillonnet, A golden gate modular cloning toolbox for plants. *ACS Synth. Biol.* **3**, 839–843 (2014).

31. C. Duggan, E. Moratto, Z. Savage, E. Hamilton, H. Adachi, C. H. Wu, A. Y. Leary, Y. Tumtas, S. M. Rothery, A. Maqbool, S. Nohut, T. R. Martin, S. Kamoun, T. O. Bozkurt, Dynamic localization of a helper NLR at the plant–pathogen interface underpins pathogen recognition. *Proc. Natl. Acad. Sci. U.S.A.* **118**, e2104997118 (2021).

32. L. Abas, C. Luschnig, Maximum yields of microsomal-type membranes from small amounts of plant material without requiring ultracentrifugation. *Anal. Biochem.* **401**, 217–227 (2010).

33. F. W. Studier, Protein production by auto-induction in high-density shaking cultures. *Protein Expr. Purif.* **41**, 207–234 (2005).

34. J. F. Steele, R. K. Hughes, M. J. Banfield, Structural and biochemical studies of an NB-ARC domain from a plant NLR immune receptor. *PLOS ONE* **14**, e0221226 (2019).

35. W. Kabsch, XDS. *Acta Crystallogr. D Biol. Crystallogr.* **66**, 125–132 (2010).

36. G. Winter, xia2: An expert system for macromolecular crystallography data reduction. *J. Appl. Cryst.* **43**, 186–190 (2010).

37. P. R. Evans, G. N. Murshudov, How good are my data and what is the resolution? *Acta Crystallogr. D Biol. Crystallogr.* **69**, 1204–1214 (2013).

38. M. D. Winn, An overview of the CCP4 project in protein crystallography: An example of a collaborative project. *J. Synchrotron Radiat.* **10**, 23–25 (2003).

39. R. Evans, M. O'Neil, A. Pritzel, N. Antropova, A. Senior, T. Green, A. Žídek, R. Bates, S. Blackwell, J. Yim, O. Ronneberger, S. Bodenstein, M. Zielinski, A. Bridgland, A. Potapenko, A. Cowie, K. Tunyasuvunakool, R. Jain, E. Clancy, P. Kohli, J. Jumper, D. Hassabis, Protein complex prediction with AlphaFold-Multimer. bioRxiv 2021.10.04.463034 [**Preprint**]. 10 March 2022. https://doi.org/10.1101/2021.10.04.463034.

40. M. Mirdita, K. Schütze, Y. Moriwaki, L. Heo, S. Ovchinnikov, M. Steinegger, ColabFold: Making protein folding accessible to all. *Nat. Methods* **19**, 679–682 (2022).

41. A. J. McCoy, R. W. Grosse-Kunstleve, P. D. Adams, M. D. Winn, L. C. Storoni, R. J. Read, Phaser crystallographic software. *J. Appl. Cryst.* **40**, 658–674 (2007).

42. P. Emsley, B. Lohkamp, W. G. Scott, K. Cowtan, Features and development of Coot. *Acta Crystallogr. D Biol. Crystallogr.* **66**, 486–501 (2010).

43. G. N. Murshudov, P. Skubák, A. A. Lebedev, N. S. Pannu, R. A. Steiner, R. A. Nicholls, M. D. Winn, F. Long, A. A. Vagin, REFMAC5 for the refinement of macromolecular crystal structures. *Acta Crystallogr. D Biol. Crystallogr.* **67**, 355–367 (2011).

44. R. A. Nicholls, M. Fischer, S. McNicholas, G. N. Murshudov, Conformation-independent structural comparison of macromolecules with ProSMART. *Acta Crystallogr. D Biol. Crystallogr.* **70**, 2487–2499 (2014).

45. E. F. Pettersen, T. D. Goddard, C. C. Huang, E. C. Meng, G. S. Couch, T. I. Croll, J. H. Morris, T. E. Ferrin, UCSF ChimeraX: Structure visualization for researchers, educators, and developers. *Protein Sci.* **30**, 70–82 (2021).

46. The PyMOL Molecular Graphics System, Version 1.2r3pre, Schrödinger, LLC.

47. M. E. Segretin, M. Pais, M. Franceschetti, A. Chaparro-Garcia, J. I. B. Bos, M. J. Banfield, S. Kamoun, Single amino acid mutations in the potato immune receptor R3a expand response to Phytophthora effectors. *Mol. Plant Microbe Interact.* **27**, 624–637 (2014).

48. D. MacLean, Besthr. Zenodo (2019).

49. I. W. Davis, A. Leaver-Fay, V. B. Chen, J. N. Block, G. J. Kapral, X. Wang, L. W. Murray, W. B. Arendall, J. Snoeyink, J. S. Richardson, D. C. Richardson, MolProbity: All-atom contacts and structure validation for proteins and nucleic acids. *Nucleic Acids Res.* **35**, W375–W383 (2007).
